# Bach2 Deficiency Leads to Spontaneous Expansion of IL-4-Producing T Follicular Helper Cells and Autoimmunity

**DOI:** 10.3389/fimmu.2019.02050

**Published:** 2019-09-04

**Authors:** Heng Zhang, Qianwen Hu, Min Zhang, Fang Yang, Cheng Peng, Zhen Zhang, Chuanxin Huang

**Affiliations:** ^1^Key Laboratory of Cell Differentiation and Apoptosis of Chinese Ministry of Education, Department of Immunology and Microbiology, Faculty of Basic Medicine, Shanghai Institute of Immunology, Shanghai Jiao Tong University School of Medicine, Shanghai, China; ^2^Shanghai Children's Medical Center, Pediatric Translational Medicine Institute, Shanghai Pediatric Congenital Heart Disease Institute, Shanghai Jiao Tong University, School of Medicine, Shanghai, China

**Keywords:** Bach2, T follicular helper cells, IL-4, autoimmunity, BCL6

## Abstract

The transcription factor Bach2 is a susceptible gene for numerous autoimmune diseases including systemic lupus erythematosus (SLE). *Bach2*^−/−^ mice can develop a lupus-like autoimmune disease. However, the exact cellular and molecular mechanisms via which Bach2 protects the hosts from developing autoimmunity remains incompletely understood. Here, we report that Bach2 ablation on T cells, but not B cells, resulted in humoral autoimmunity, and this was associated with expansion of T follicular helper (Tfh) cells and abnormal germinal centers. Bach2 was down-regulated in Tfh cells and directly suppressed by the Tfh-defining transcription factor BCL6. Mechanistically, Bach2 directly suppresses the transcription of Cxcr5 and c-Maf, two key regulators of Tfh cell differentiation. Bach2-deficient Tfh cells were skewed toward the IL-4-producing subset, which induced IgG1 and IgE isotype switching of B cells. Heterozygous Bcl6 deficiency reduced the formation of germinal center and autoantibodies, and ameliorated the pathology in Bach2-deficient mice. Our findings identify Bach2 as a crucial negative regulator of Tfh cells at steady state and prove that Bach2 controls autoimmunity in part by restraining accumulation of pathogenic Tfh cells.

## Introduction

The transcription factor Bach2 belongs to the BTB and Cap'n'collar (CNC) gene family and functions within multiple innate and adaptive lineages to control immune responses (1). Genetic polymorphisms within the *BACH2* gene locus in human are associated with numerous autoimmune and allergic diseases including asthma (2), vitiligo (3), multiple sclerosis (4), type I diabetes (5), and systemic lupus erythematosus (SLE) (6). *Bach2*^−/−^ mice developed autoantibodies and spontaneous lethal inflammatory diseases (7), suggesting an essential role of Bach2 in controlling autoimmunity. Bach2 is highly expressed in GC B cells and promotes antibody class switching and suppresses plasma cell differentiation (8, 9). Bach2 is also expressed by T cells, and directs T helper (Th) cell differentiation, homeostasis, and effector functions (1). Bach2 constrains full effector differentiation within Th1, Th2, and Th17 cells *in vitro* (7). Bach2 maintains Th cells in a naïve state by suppressing effector memory-related genes (10). Bach2 promotes Foxp3^+^ regulatory T (Treg) cell differentiation and enhances Treg-mediated immunity (7, 11). More recently, we reported that SUMO-specific protease 3 (SENP3) act as a pivotal regulator of Treg cells by controlling the SUMOylation and nuclear localization of Bach2 (12). In addition, Bach2 suppresses the differentiation of Th2 cells, and *Bach2*-deficient mice develop lethal lung-specific Th2-type allergic inflammation (13, 14). These findings help explain the emergence of Bach2 as a key suppressor of autoimmunity.

T follicular helper (Tfh) cells, a unique subset of CD4 helper T cells, are specialized in providing cognate help to B cells to form GCs. Within GCs, Tfh cells interact with B cells through the co-stimulatory and co-inhibitory molecules, including CD40L, ICOS, and programmed cell death protein 1 (PD-1), to facilitate the selection and maturation of high-affinity B cells (15, 16). However, excessive Tfh cells impair positive selection by reducing competition among B cells for T cell help, thus yielding a lower threshold for selection that allows for emergence of self-reactive clones. Aberrant expansion of Tfh cells results in pathogenic autoantibodies and is frequently associated with autoimmune diseases, including SLE (17–19). Numerous mouse studies have supported a causal role of Tfh cells in autoimmune diseases (20–22). In addition, increased blood memory Tfh cells were frequently observed in human autoimmune diseases such as SLE, and positively correlated with serum autoantibody titers and disease severity (23, 24), suggesting an important role of Tfh cells in the pathogenesis of these diseases. Tfh cells are proposed to be potential therapeutic targets in human autoimmune diseases.

Tfh cells are generally considered to be of a separate Th cell lineage and arise from naive CD4^+^ T cells with sequential steps in response to T-cell-dependent antigen (15, 16). Naive CD4^+^ T cells are primed by dendritic cells and up-regulates the chemokine (C-X-C motif) receptor CXCR5, which enables them to migrate into B cell follicles to further differentiate into Tfh cells. However, Tfh cells can also be generated from the conversion of other effector T cells under the conditions of chronic and sustained antigenic stimulation (25, 26). Tfh cell differentiation is tightly controlled by numerous lineage-specific transcription factors including B cell lymphoma 6 (BCL6), c-Maf, and Blimp1 (27, 28). BCL6 is a master transcription factor of Tfh cells, and Tfh cell differentiation is completely abrogated in *Bcl6*-deficient CD4^+^ T cells (29–31). c-Maf acts as a positive regulator of Tfh cell differentiation in mice and humans, and induces the expression of many Tfh-related genes including *Cxcr5* and *IL-21* (32–34). Interestingly, some regulators of Tfh cell differentiation, such as transcription factor Ets1 (35), are encoded by human autoimmunity-prone genes, and their deficiency causes Tfh-driven humoral autoimmune diseases in mice.

In this study, we report that genetic deletion of *Bach2* in T cells, but not in B cells, recapitulated the lupus-like autoimmunity in *Bach2*^−/−^ mice. *Bach2* deficiency in T cells results in spontaneous accumulation of IL-4-producing Tfh cells. We also provide the evidence that Bach2 controls autoimmunity in part by restraining aberrant Tfh cell formation.

## Materials and Methods

### Mice

*Bach2*^fl/fl^ mice (ES Clone ID: *EPD0689_1_H02*) were generated at the Wellcome Trust Sanger Institute and the neo cassette was removed after crossing with FLPe transgenic mice (The Jackson Laboratory). Cd4-Cre, Cd19-Cre, and ERT2-Cre transgenic mice were from The Jackson Laboratory. *Bcl6*^fl/fl^ mice are described previously (36). All mice were crossed on a C57BL/6J background. All mice were maintained in a specific pathogen-free (SPF) facility, and all animal experiments were in accordance with protocols approved by the Institutional Animal care and Use Committee (IACUC) of Shanghai Jiao Tong University, School of Medicine.

### Flow Cytometry and Antibodies

Single-cell suspensions of spleens, MLNs, and PPs were prepared from fresh tissues by standard procedures and surface-stained in FACS buffer with monoclonal antibodies. The following antibodies were obtained from eBioscience, BD PharMingen, or BioLegend: anti-B220-PE, FITC, BV650 or APC (RA3-6B2), anti-Fas PE-Cy7 (Jo2), anti-CD38-PE or PerCP/Cy5.5, anti-GL7-FITC (GL7), anti-IgM-APC (II/41), anti-IgG1-FITC (RMG1-1), anti-IgE-FITC (RME-1), anti-CD86-APC (GL1), anti-ICOS-FITC (7E.17G9), anti-CD44-APC (1M7), anti-CD62L-FITC (MEL-14), anti-PD1-PE or FITC (J43), and anti-CD4-APC or APC-CY7 (GK1.5). CXCR5 was stained with biotinylated anti-CXCR5 (clone 2G8) and streptavidin-conjugated PE, APC, or PE/CY7 (BD Biosciences). For intracellular staining, cells were fixed and permeabilized with “Foxp3 staining buffer set” (eBioscience) following the manufacturer's protocol and then stained with anti-Foxp3-PE (FJK-16s, ebiosciences) and anti-BCL6-PerCP/CY5.5. To identify cytokine secretion, lymphocytes were routinely prepared and stimulated with PMA (phorbol 12-myristate 13-acetate, 20 ng/ml, Invitrogen) and inomycin (1 μg/ml, Invitrogen) in the presence of Golgi-Plug (1 μg/ml, Invitrogen) for 5 h. Cells were stained with anti-CD4 antibodies, followed by permeabilization in Fix/Perm buffer (BD, Pharmingen), and intracellular staining in Perm/Wash buffer (BD Pharmingen) with anti-IL-4-PE (11b11) and anti-IFN-gamma-FITC (XMG1.2). NP-PE was from Biosearch Technologies. FACS analysis was performed on a Fortessa II (BD), and data were analyzed with FlowJo software (Tree Star).

### Histology

Kidneys were fixed in 10% neutral buffered formalin, embedded in paraffin, sectioned at 7 μm, and stained with hematoxylin and eosin. Immunohistochemistry was performed as described previously (9). Briefly, after antigen retrieval and pretreatment with 3% hydrogen peroxide, tissue sections (7 μm) were then incubated with biotin-conjugated PNA (Vector Laboratories) overnight at 4°C, followed by streptavidin–alkaline phosphatase and detected by the Alkaline Phosphatase Substrate Kit III (Vector Laboratories). The sections were then boiled for 10 min, incubated with biotin-conjugated anti-B220 (RA3-6B2, Caltag metsystems), followed by streptavidin-HRP and developed using DAB kit. To visualize IgG position in the glomeruli, kidney sections (7 μm) were incubated with blocking buffer and stained with HRP-conjugated anti-mouse IgG antibody (Jackson ImmunoResearch), followed by streptavidin-HRP and developed using DAB kit. Images were taken using a color camera (AxioCam; Carl Zeiss Microimaging) and were analyzed using Axiovision software (Carl Zeiss Microimaging).

### Immunization and Tamoxifen Treatment

Mice were immunized intraperitoneally (i.p.) with SRBCs (1 × 10^8^ cells per mouse). Tamoxifen (80 mg/kg, Sigma) in corn oil (Sigma) was given by oral gavage on day 8 post immunization to induce the activity of the Cre recombinase. Mice were analyzed on day 5 after tamoxifen treatment.

### Mixed Bone Marrow Chimera

To determine the intrinsic role of Bach2, a mixture of congenic CD45.1^+^ wild-type bone marrow cells (80%) together with CD45.2^+^ tested bone marrow cells (20%) either from WT or *Bach2*^Δ*CD*4^ mice were transferred intravenously into lethally irradiated (5.5 Gy, twice) CD45.1^+^ WT recipient mice. After at least 8 weeks of bone marrow reconstitution, the recipients were subjected for analysis.

### RNA Sequencing and Data Analysis

A total of 1–3 × 10^4^ Tfh cells (CD4^+^B220^−^CD44^+^PD-1^+^CXCR5^+^) were sorted from the MLNs of WT and *Bach2*^fl/fl^Cd4-cre mice using a FACSAria II cell sorter (BD Biosciences) at the core facility of Shanghai Institute of Immunology. cDNA was synthesized and amplified using the SMARTer Ultra Low RNA Kit for Illumina Sequencing (Clontech). The library for mRNA sequencing was generated using Illumnia TruSeq Preparation Kit (RS-122-2001) and sequenced using Illumnia Nextseq500 sequencer. The mapping rate was 96% overall across all the samples in the dataset. HTSeq was used to quantify the gene expression counts from Tophat2 alignment files. Differential expression analysis was performed on the count data using R package DESeq2. *P* values obtained from multiple tests were adjusted using Benjamini-Hochberg correction. Significantly differentially expressed genes are defined by a Benjamini-Hochberg corrected *P* value < 0.01 and fold change > 2.

### Enzyme-Linked Immunosorbent Assay (ELISA) and Detection of Autoantibodies

Serum titers of immunoglobulin subclasses were determined by specific ELISA kits (SouthernBiotech) according to the manufacturer's protocol. To detect anti-dsDNA autoantibodies in sera, high-binding ELISA plates were coated overnight with 2 μg/ml dsDNA from calf thymus (Sigma-Aldrich). Coated plates were blocked with 1% BSA and 0.5% gelatin in TBS for 2 h at room temperature, and diluted samples were incubated overnight at 4°C in TBS with 1% BSA. Bound anti-dsDNA antibodies were detected with AP-conjugated anti-mouse IgG (Jackson ImmunoResearch) and streptavidin-HRP (Bioresearch) followed by TMB substrate solution (eBioscience). Absorbance was measured at 450 nm. Serum titers of anti-ANA antibodies were determined by ANA Hep Screen ELISA kit (Demeditec) according to the manufacturer's protocol.

### Naive CD4^+^ T Cell Isolation and Differentiation *in vitro*

Naive CD4^+^ T (CD44^low^CD62L^high^CD25^−^) cells were purified using a CD4^+^ naive T cell negative isolation kit according to the manufacturer's protocol (STEMCELL Technologies). The *in vitro* differentiation experiments were performed as previously described. Naive CD4^+^ T cells were stimulated with immobilized anti-CD3 (5 μg/ml; 145-2C11; eBioscience) and anti-CD28 (5 μg/ml; 37.51; eBioscience) for 2 days. Then, the cells were washed and transferred to a new plate and further expanded in medium with hIL-2 (50 U/ml, R&D Systems) for 2 days. For Tfh-like cell differentiation, naive CD4^+^ T cells were activated with anti-CD3 and anti-CD28 as above and treated with 20 ng/ml IL-6 (R&D Systems), 20 ng/ml IL-21 (R&D Systems), 10 μg/ml anti-IL-4 (11B11, eBioscience), 10 μg/ml anti-IFN-γ (XMG1.2, eBioscience), and 20 μg/ml anti-TGF-β (1D11, R&D Systems) for 4 days.

### RT-qPCR

Total RNA was prepared with Trizol (invitrogen) and cDNA was synthesized using Superscript reverse transcriptase and random primers (Invitrogen). Quantitative PCR (qPCR) was performed using Power SYBR Green PCR master mix (Vazyme). The sequences of gene-specific primers are listed in the Supplementary Information. All reactions were performed in triplicate and results were calculated by the change-in-threshold (2^−ΔΔ*CT*^) method with *Act*β as housekeeping reference gene.

### ChIP-qPCR

Spleen naive CD4^+^ T cells were stimulated with immobilized anti-CD3 (5 μg/ml; 145-2C11; eBioscience) and anti-CD28 (5 μg/ml; 37.51; eBioscience) antibodies for 2 days before analysis. Human Tfh cells were isolated from tonsils as described previously (37). Cells were cross-linked with 1% formaldehyde and neutralized with 0.125 M glycine. Cell lysates were sonicated to 300–500 bp and immunoprecipitations were performed with anti-BCL6 (N-3; Santa Cruz Biotechnology Inc.), anti-BACH2 (9), or IgG as a control. After complete washing, immunoprecipitated DNA was eluted in elution buffer and reversely cross-linked at 65°C overnight. DNA was purified and quantified by real-time PCR. Gene-specific primers are listed in Table S1.

### Statistical Analysis

Student's *t*-test was performed for statistical analysis. The software GraphPad Prism 5 was used for this analysis. *P* values higher than 0.05 are considered to be not significant.

## Results

### *Bach2* Ablation in T Cells Is Sufficient to Recapitulate the Autoimmunity in *Bach2^−/−^* Mice

**Figure 1:**
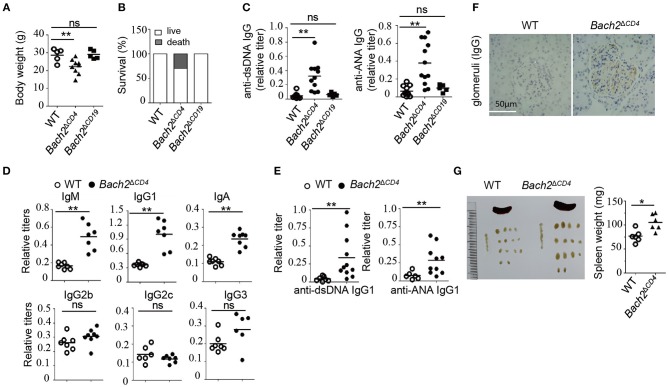
Loss of Bach2 in T cells causes humoral autoimmunity. **(A,B)** Body weight **(A)** and survival rate **(B)** of wild-type (WT), *Bach2*^Δ^^CD4^, or *Bach2*^Δ*CD*19^ mice at 5 months of age. **(C)** Titer of anti-dsDNA antibodies and anti-nuclear antibodies (ANA) in the sera of WT, *Bach2*^Δ^^CD4^, or *Bach2*^Δ*CD*19^ mice at 2–3 months of age. **(D)** Titers of immunoglobulins in sera of WT and *Bach2*^Δ^^CD4^ mice at 2–3 months of age. **(E)** Titers of anti-dsDNA and ANA IgG1 in the sera of WT and *Bach2*^Δ^^CD4^ mice at 2–3 months of age. **(F)** Immunochemistry of IgG deposit in renal glomeruli. **(G)** Representative images (left) of spleen and lymph nodes of WT and *Bach2*^Δ^^CD4^ mice at 5 months of age. The weight of spleens from indicated mice is shown at the right. Each symbol represents one mouse; small horizontal lines indicate the mean; ns, not significant; ^*^P < 0.05 and ^**^P < 0.01 (two-tailed *t*-test).

*Bach2*^−/−^ mice have been known to develop autoantibodies and age-related lethal autoimmunity (7). To determine which types of immune cells play key roles in disease development, we generated cell-type-specific *Bach2*-deficient mice where *Bach2* was deleted in CD4^+^ T cells (*Bach2*^Δ*CD*4^) or B cells (*Bach2*^Δ*CD*19^). *Bach2*^Δ*CD*4^ mice were undistinguishable from the *Cd4*-Cre mice (designated WT) littermates by 2 months of age. From 3 months of age, *Bach2*^Δ*CD*4^ mice spontaneously developed a progressive wasting disease that resulted in diminished survival compared to WT mice (Figures 1A,B). Two- to 3-month-old *Bach2*^Δ*CD*4^ mice generated higher concentrations of antibodies to double-stranded DNA (anti-dsDNA) and nuclear antigen (anti-ANA) in their serum (Figure 1C), which are the hallmarks of autoimmune diseases in humans. In contrast, these abnormalities were not detected in *Bach2*^Δ*CD*19^ mice (Figures 1A–C). Two- to 3-month-old *Bach2*^Δ*CD*4^ mice contained significantly higher amounts of serum IgM, IgG1, and IgA compared to age-matched WT controls (Figure 1D). However, the titers of serum IgG2b, IgG2c, and IgG3 were comparable between the two groups (Figure 1D). We also detected higher titers of anti-dsDNA and anti-ANA IgG1, but not other IgG2 subclasses, in the serum of these *Bach2*^Δ*CD*4^ mice (Figure 1E and data not shown). Aged *Bach2*^Δ*CD*4^ mice displayed prominent IgG deposits in kidney glomeruli (Figure 1F), and spontaneously developed splenomegaly and lymphadenopathy (Figure 1G). These abnormalities became prominent when they reached 5–6 months of age and showed signs of illness. In summary, these results demonstrate that Bach2 in T cells is essential to prevent humoral autoimmunity in mice.

### *Bach2* Deficiency Results in Excessive Tfh Cells and Aberrant GC B Cells in Mesenteric Lymph Nodes and Peyer's Patches

**Figure 2:**
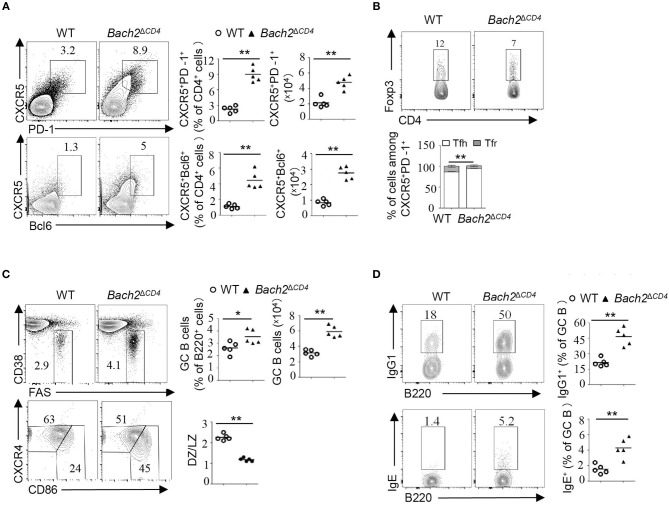
*Bach2*^Δ^^CD4^ mice display excessive Tfh cells and aberrant GC B cells in the MLNs. Lymphocytes from the MLNs of WT and *Bach2*^Δ^^CD4^ mice at 2–3 months of age were subjected for analysis. **(A)** Representative flow cytometry plots, frequency quantification, and absolute number of CXCR5^+^PD-1^+^ and CXCR5^+^Bcl6^+^ T follicular cells (gated on CD4^+^B220^−^ cells). **(B)** Representative flow cytometry plots and frequency quantification of Tfh (Foxp3^−^) and Tfr (Foxp3^+^) cells among CXCR5^+^PD-1^+^ CD4 T cells. **(C)** Representative flow cytometry plots, frequency quantification, and absolute number of CD38^lo/−^Fas^+^ GC B cells among splenic live B220^+^ cells. Quantification of the ratio of CD86^hi^CXCR4^lo^ light zone (LZ) to CD86^lo^CXCR4^hi^ dark zone (DZ) B cells among GC B cells (bottom). **(D)** Representative flow cytometry plots and frequency quantification of IgG1^+^ and IgE^+^ cells among splenic GC B cells. All data were from at least two independent experiments. Each symbol represents one mouse, and small horizontal lines indicate the mean. ns, not significant; ^*^P < 0.05 and ^**^P < 0.01 (two-tailed *t*-test).

Abnormal Tfh cell responses can lead to the development of autoimmunity (19, 26). We next examined whether Tfh cells and GCs were altered in *Bach2*^Δ*CD*4^ mice. GCs are chronically induced in the mesenteric lymph nodes (MLNs) and Peyer's patches, as a result of continuous B cell stimulation by a wide range of microbiota and food-derived antigens. For the next analyses, we used 8–10-week-old *Bach2*^Δ*CD*4^ mice before they developed lymphoproliferative disease. The MLNs from *Bach2*^Δ*CD*4^ mice had normal cellularity, but a lower proportion of CD4^+^ T cells when compared to the WT counterparts (Figure S1A). Consistent with previous reports, *Bach2*^Δ*CD*4^ mice contained a higher frequency of CD44^hi^ active/memory fraction in the CD4^+^ compartment within the MLNs (Figure S1B). However, *Bach2*^Δ*CD*4^ mice had a significantly higher frequency and absolute number of follicular T cells (CXCR5^+^PD-1^+^ or CXCR5^+^Bcl6^+^) among CD4^+^ T cells than WT mice (Figure 2A). We next sought to determine whether expansion of follicular T cells in *Bach2*^Δ*CD*4^ mice was selective to Tfh (CXCR5^+^PD-1^+^Foxp3^−^) and/or Tfr (CXCR5^+^PD-1^+^Foxp3^+^) cells. We found that among CXCR5^+^PD-1^+^ follicular T cell compartments in *Bach2*^Δ*CD*4^ mice, the ratio of Tfh to Tfr cells was shifted significantly toward the Tfh cell subset (Figure 2B), suggesting an expansion of Tfh cells to a larger extent than that of Tfr cells. Ablation of *Bach2* in T cells has been shown to decrease Foxp3^+^ Treg cells (7). Indeed, the percentage of Treg cells tended to decrease in the MLNs of *Bach2*^Δ*CD*4^ mice, although with no significance (Figure S1C). The absolute number of Treg cells in the MLNs of *Bach2*^Δ*CD*4^ mice was significantly reduced because of their decreased CD4^+^ T cell compartment (Figure S1C). Interestingly, we found that the frequencies of Tfr cells among Foxp3^+^ CD4^+^ Treg cells and Tfh cells among Foxp3^−^ CD4^+^ T cells increased by about two- and four-fold in the MLNs of *Bach2*^Δ*CD*4^ mice compared to WT mice, respectively (Figure S1D). The absolute numbers, however, were significantly elevated only for Tfh but not Tfr cells in *Bach2*^Δ*CD*4^ mice (Figure S1D). Thus, the Tfr/Tfh ratio, a critical factor dictating the magnitude of antibody production, was reduced in *Bach2*^Δ*CD*4^ mice due to greater expansion of Tfh cells relative to Tfr cells.

We then investigated whether excessive Tfh cells induced abnormal GCs in the MLNs of *Bach2*^Δ*CD*4^ mice. *Bach2*^Δ*CD*4^ mice had a significantly higher frequency and absolute number of Fas^+^CD38^lo^ GC B cells in the MLNs when compared to WT mice (Figure 2C). Moreover, *Bach2*^Δ*CD*4^ mice had a greater proportion of CXCR4^lo^CD86^hi^ light zone cells among GC B cells (Figure 2C). The proportions of IgG1^+^ and IgE^+^ GC B cells were significantly increased in the MLNs of *Bach2*^Δ*CD*4^ mice (Figure 2D), indicating that Bach2 in Tfh cells affects antibody isotype switch within B cells. Similarly, excessive Tfh cells and deregulated GCs were observed in the Peyer's patches of *Bach2*^Δ*CD*4^ mice (Figure S2). To further determine the cell-intrinsic role of Bach2 in constraining Tfh cells, we generated mixed bone marrow chimeras by reconstituting lethally irradiated CD45.1^+^ WT mice with a 4:1 ratio mixture of bone marrow cells from CD45.2^+^ *Bach2*-deficient and WT donor mice, respectively (Figure S3). Eight weeks after transplantation, we observed that CD45.2^+^ *Bach2*-deficient CD4^+^ T cells developed more Tfh cells compared to CD45.2^+^ WT CD4^+^ T cells in the MLNs and Peyer's patches (Figure S3), indicating that Bach2 regulates Tfh cell formation intrinsically. In conclusion, Bach2 in T cells is critical to limit Tfh cell differentiation and regulate the GC responses in chronic GCs.

### *Bach2* Deficiency in T Cells Enhances the Formation of Spontaneous GCs in the Spleen

**Figure 3:**
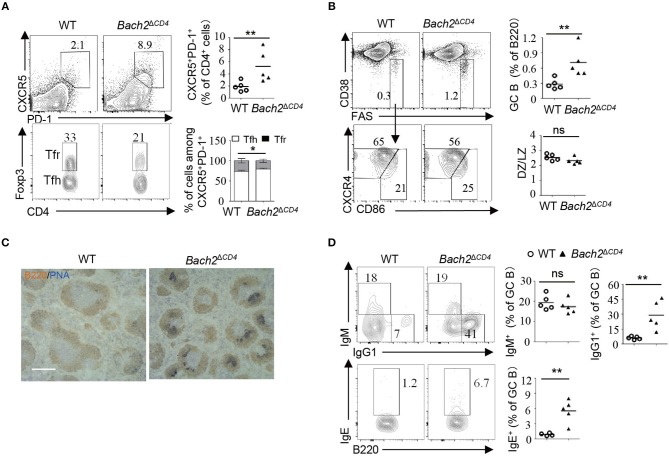
*Bach2*^Δ^^CD4^ mice develop spontaneous GCs in the spleens with age. The lymphocytes from the spleens of WT and *Bach2*^Δ^^CD4^ mice at 3–5 months of age were subjected for analysis. **(A)** Representative flow cytometry plots and frequency quantification of splenic CXCR5^+^PD-1^+^ T follicular cells (gated on CD4^+^B220^−^ cells) (top). Representative flow cytometry plots and frequency quantification of Tfh (Foxp3^−^) and Tfr (Foxp3^+^) cells among CXCR5^+^PD-1^+^ CD4 T cells (bottom). **(B)** Representative flow cytometry plots and frequency quantification of GC B cells among splenic live B220^+^ cells. Quantification of the ratio of LZ to DZ B cells among GC B cells (bottom). **(C)** Representative PNA staining of splenic sections from WT and *Bach2*^Δ^^CD4^ mice (scale bars, 1 mm). Blue, PNA; brown, B220. **(D)** Frequencies of IgM^+^, IgG1^+^, and IgE^+^ cells among GC B cells. All data were from at least two independent experiments. Each symbol represents one mouse, and small horizontal lines indicate the mean. ns, not significant; ^*^P < 0.05 and ^**^P < 0.01 (two-tailed *t*-test).

Spontaneous GCs are induced without immunization or infection, and contribute to steady-state antibody production. Aberrant spontaneous GCs produce pathogenic autoantibodies and have been described in many autoimmune diseases such as SLE (21). We next explored whether Bach2 deficiency in T cells affects the generation of spontaneous GCs. We found that 4–5-month-old *Bach2*^Δ*CD*4^ mice had significantly higher frequencies of CXCR5^+^PD-1^+^ follicular T cells in their spleens (Figure 3A). Among the follicular T cell compartment in *Bach2*^Δ*CD*4^ mice, the Tfh to Tfr cell ratio shifted significantly toward the Tfh cell subset in their spleens (Figure 3A). Furthermore, the frequency of GC B cells was significantly elevated in the spleens of *Bach2*^Δ*CD*4^ mice compared to WT mice (Figure 3B). Immunohistochemical staining further revealed that the spleens of *Bach2*^Δ*CD*4^ mice contained considerably more and larger peanut agglutinin (PNA)-positive GCs than did their WT counterparts (Figure 3C). Although the frequencies of dark zone B cells among GC B cells in the spleen of *Bach2*^Δ*CD*4^ mice were equivalent to that of WT mice (Figure 3B), the proportion of IgG1^+^ and IgE^+^ cells was significantly higher (Figure 3D). Interestingly, the accumulation of Tfh cells and GC B cells was not observed in the spleens of young *Bach2*^Δ*CD*4^ mice at 6–8 weeks of age (data not shown). In conclusion, Bach2 in T cells is required to limit the formation of spontaneous GCs under steady state.

### Bach2 Is Down-Regulated During Tfh Cell Differentiation and Directly Suppressed by BCL6

**Figure 4:**
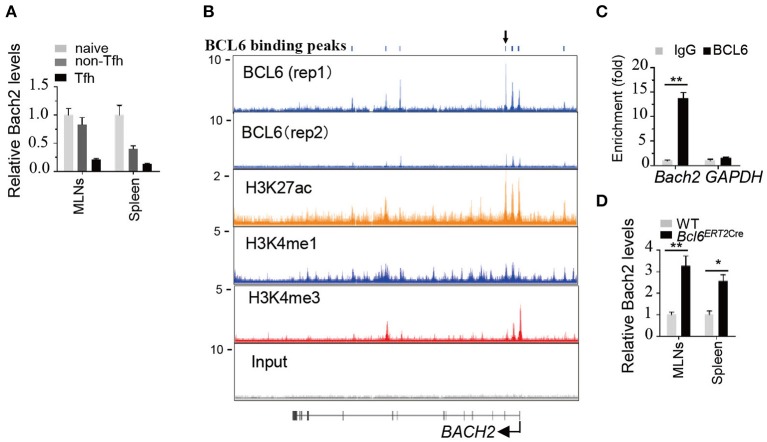
*Bach2* is down-regulated during Tfh cell differentiation and directly suppressed by BCL6. **(A)** Relative *Bach2* mRNA abundance in naïve (CD44^low^CD62L^high^), non-Tfh (CD44^high^CD62L^low^CXCR5^−^PD1^−^), and Tfh cells isolated from the MLNs and/or spleens of WT mice at 2 months of age, determined by RT-qPCR. **(B)** The illustration depicting ChIP-seq tracks of BCL6 and indicated histone marks at the *BACH2* gene in primary human Tfh cells. BCL6 binding peaks are shown at the top. **(C)** ChIP-qPCR analysis of BCL6 binding peak indicated by arrow in **(B)**. The fold enrichment was calculated relative to IgG. Data are mean ± s.e.m. **(D)** The relative mRNA expression levels of *Bach2* in Tfh cells sorted from the MLNs and spleens of 2-month-old WT and *Bcl6*^*ERT*2*Cre*^ mice 5 days post tamoxifen treatment. Data are mean ± s.e.m. of two mice per group. ^*^P < 0.05 and ^**^P < 0.01 (two-tailed *t*-test).

A previous study showed that naive CD4^+^ T cells express higher levels of Bach2 compared to effector/memory CD4^+^ cells (30). To better understand the role of Bach2 in Tfh cells, we first examined the mRNA abundance of Bach2 during Tfh cell differentiation. Quantitative RT-PCR revealed that *Bach2* mRNA abundance was markedly reduced during Tfh cell differentiation (Figure 4A). Then, we sought to figure out the mechanism by which Bach2 is suppressed in Tfh cells. The transcriptional repressor BCL6 is highly expressed in Tfh cells and acts as a master regulator of Tfh differentiation (15–17). Therefore, we hypothesized that Bach2 may be a direct target of BCL6. By analyzing published chromatin immunoprecipitation sequencing (ChIP-Seq) of human primary tonsillar Tfh cells (35), we identified multiple BCL6 binding peaks at the BACH2 gene promoter and gene body (Figure 4B). The binding of BCL6 at the *BACH2* promoter region was confirmed by ChIP followed by quantitative PCR (Figure 4C). To investigate whether BCL6 represses Bach2 expression, WT and *Bcl6*^*ERT*2*Cre*^ mice were immunized i.p. with SRBCs, a T-cell-dependent antigen, to induce Tfh cell response in the spleen. On day 8 after immunization, mice were administrated with one dose of tamoxifen by oral gavage to induce Cre activity for *Bcl6* deletion in Tfh cells. Acute deletion of *Bcl6* in Tfh cells led to an increase of *Bach2* mRNA abundance (Figure 4D). Collectively, these results demonstrate that BCL6 directly binds to Bach2 and suppresses its transcription.

### Bach2 Represses Tfh-Related Genes Including *C-Maf* and *Cxcr5*

**Figure 5:**
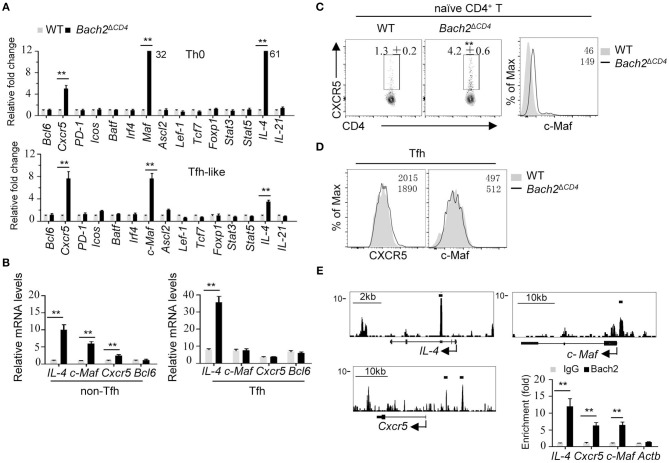
Bach2 negatively regulates the mRNA abundance of *Cxcr5, Maf*, and *IL-4*. All cells were isolated from 2-month-old mice for analysis. **(A)** Relative mRNA levels of indicated genes in splenic naïve CD4^+^ cells (CD44^low^CD62L^high^) cultured in Th0 or Tfh-like cell condition. Data were from two independent experiments and represented as fold changes relative to WT cells after normalization with *Actb*. Numbers adjacent to black bars indicate fold in each. **(B)** RT-qPCR analysis of mRNA levels of indicated genes in WT and *Bach2*-*deficient* non-Tfh (CD44^high^CXCR5^−^PD1^−^) and Tfh cells. Data were represented as up-regulation relative to WT non-Tfh cells after normalization with *Actb*. **(C)** The frequency of CXCR5^+^ cells and c-Maf protein abundance in naïve CD4^+^ T cells from the MLNs. **(D)** Flow cytometric analysis of CXCR5 and c-Maf protein abundance in Tfh cells. **(E)** The illustration of ChIP-seq tracks of Bach2 at indicated genes in CD4^+^ T cells (left). Bach2-bound sites were indicated by black lines. ChIP-qPCR analysis of Bach2 binding at indicated genomic loci. The fold enrichment was calculated relative to IgG. Data are mean ± s.e.m. of two independent experiments from at least three mice per group. ^**^P < 0.01 (two-tailed *t*-test).

To gain further insight into the mechanisms by which Bach2 inhibits Tfh cell differentiation, we compared the mRNA abundance of a group of key molecules known to be crucial for the differentiation of Tfh cells in WT and *Bach2*-deficient naive CD4^+^ T cells activated *in vitro* under unbiased conditions (Th0) or conditions that promote the differentiation of Tfh cell-like (Tfh-like) cells. *Bach2* deficiency promotes activated CD4^+^ T cells to express high level of *IL-4* (Figure 5A), in agreement with a previous report (13, 14). Interestingly, the mRNA levels of *Cxcr5* and c-*Maf* were up-regulated by about 6- and 32-fold in *Bach2*-deficient vs. WT CD4^+^ T cells cultured in Th0 cell condition, respectively (Figure 5A). Moreover, the mRNA levels of *Cxcr5* and c-*Maf* were increased by about seven-fold in *Bach2*-deficient CD4^+^ T cells cultured under Tfh-like cell conditions (Figure 5A). However, the expression levels of other Tfh cell-related genes, including *Bcl6, Batf*, and *Icos*, were largely unchanged, suggesting that Bach2 may suppress Tfh differentiation mainly *via* repression of *Cxcr5* and c-*Maf*. In line with this, *Bach2*-deficient non-Tfh (CD4^+^CD44^high^CXCR5^−^PD1^−^) cells expressed higher mRNA levels of c-*Maf, Cxcr5*, and *IL-4*, but not *Bcl6* (Figure 5B). Unexpectedly, Bach2 appeared not to affect the expression of c-*Maf* and *Cxcr5* in Tfh cells (Figure 5B), and this is likely due to low abundance of Bach2 in these cells (Figure 4A). Finally, we observed that *Bach2*-deficient naïve CD4^+^ T cells contained a greater proportion of CXCR5^+^ cells and expressed higher c-Maf protein abundance *in vivo* (Figure 5C). The protein expression of CXCR5 and c-Maf was comparable between WT and *Bach2*-deficient Tfh cells (Figure 5D).

We next investigated the molecular mechanism by which Bach2 suppresses *Cxcr5* and c-*Maf* transcription. Bach2 is known to act primarily as a transcription repressor. By analyzing published Bach2 ChIP-Seq data in activated CD4^+^ T cells (13), we found that Bach2 was recruited to the intron region of *IL-4*, the promoter region of c-*Maf* and upstream of the promoter of *Cxcr5* (Figure 5E). The binding of Bach2 at these loci were further confirmed by ChIP-qPCR in activated CD4^+^ T cells (Figure 5E), indicating that Bach2 negatively and directly regulates *IL-4*, c-*Maf*, and Cxcr5. Interestingly, Bach2 bound to the *IL-4* gene, but not *Cxcr5* and c-*Maf* genes in Tfh-like cells (Figure S4), consistent with the observation that WT and Bach2-deficient Tfh cells expressed the similar levels of Cxcr5 and c-Maf (Figures 5B,D).

### *Bach2* Deficiency Promotes Tfh Cells to Skew Toward IL-4-Producing Subset

**Figure 6:**
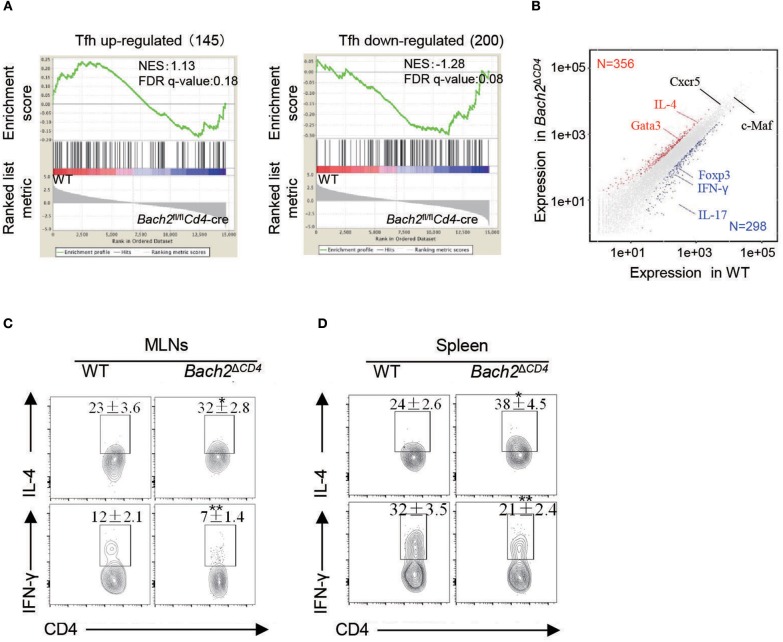
*Bach2*-deficient Tfh cells are skewed IL-4-producing subset. Two- to 3-month-old mice were used for analysis. **(A)** GSEA analysis of gene signatures up-regulated or down-regulated in Tfh cells relative to their expression in non-Tfh cells, from published data (GEO accession code: GSE21379). **(B)** Scatterplot of genes up-regulated (red) or down-regulated (blue) in *Bach2*-deficient Tfh cells vs. WT cells. Select genes of interest are labeled. **(C,D)** Representative flow cytometry plots and quantification of IL-4 and IFN-γ-secreting Tfh subsets (gated in CXCR5^+^PD-1^+^ T follicular cells) in the MLNs **(C)** and spleens **(D)**. Data are mean ± s.e.m. of two independent experiments from at least three mice per group. ^*^P < 0.05 and ^**^P < 0.01 (two-tailed *t*-test).

To further elucidate the molecular mechanisms by which Bach2 regulates Tfh cell differentiation *in vivo*, we performed RNA sequencing to compare the transcriptome between WT and *Bach2*-deficient Tfh cells. The global gene expression between WT and *Bach2*-deficient Tfh cells were highly concordant (Pearson *r*^2^ = 0.92, three replicates for each group), and key molecules known to be critical for Tfh cell differentiation, including *Cxcr5* and *c-Maf*, had similar expression in the two groups of Tfh cells. Then, we selected genes from published datasets (GEO accession codes GSE21379) that are up-regulated and down-regulated in Tfh cells relative to their expression in non-Tfh cells (38), for gene set enrichment analysis (GSEA) with our data. This analysis revealed that *Bach2-*deficient Tfh cells did not show significant enrichment for the genes up-regulated or down-regulated in the Tfh lineage (Figure 6A), further suggesting that *Bach2*-deficient Tfh cells are “true” Tfh cells. Only 356 genes were significantly (*p*_adj_ < 0.01; change in expression ≥2-fold) up-regulated and 298 genes were down-regulated in *Bach2*-deficient Tfh cells (Figure 6B). Among them, we found that *Bach2*-deficienct Tfh cells expressed higher *IL-4*, but lower IFN-γ, IL17-f, and Foxp3 at mRNA levels (Figure 6B). Moreover, the Th2 master regulator *Gata3* was also increased in *Bach2*-deficienct Tfh cells (Figure 6B). Flow cytometry further revealed that *Bach2*-deficient Tfh cells contained a higher proportion of IL-4-secreting cells, but a lower frequency of IFN-γ-secreting cells, in both the MLNs and spleens (Figures 6C,D), indicating that *Bach2* deficiency promotes Tfh cells to skew toward IL-4- producing subset.

### Heterozygous *Bcl6* Deficiency Reduces Autoantibodies and Attenuates Pathology in *Bach2^ΔCD4^* Mice

**Figure 7:**
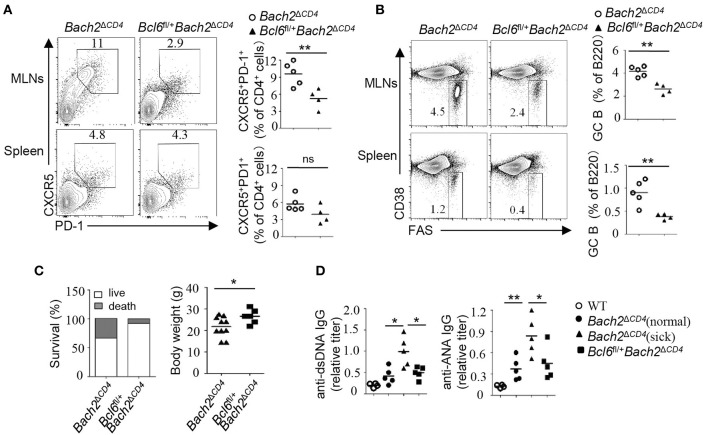
The *Bcl6* heterozygosity significantly reduced autoantibodies and pathology. **(A,B)** The frequencies of Tfh and GC B cells in the MLNs and spleens from *Bach2*^Δ^^CD4^ and *Bcl6*^fl/+^*Bach2*^Δ^^CD4^ mice. **(C)** Body weight and survival of *Bach2*^Δ^^CD4^ and *Bcl6*^fl/+^*Bach2*^Δ^^CD4^ mice. **(D)** Titers of anti-dsDNA and anti-ANA antibodies in sera from *Bach2*^Δ^^CD4^ and *Bcl6*^fl/+^*Bach2*^Δ^^CD4^ mice. All mice were analyzed at 5–6 months of age. Each symbol represents one mouse; small horizontal lines indicate the mean. ns, not significant; ^*^P < 0.05 and ^**^P < 0.01 (two-tailed *t*-test).

Autoantibodies result from a breakdown of tolerance mechanisms during B cell development, from T-cell-dependent or -independent B cell activation, or as a consequence of aberrant GC reactions. BACH2 has been shown to regulate many aspects of T cell differentiation and effector functions (1). Therefore, it is important to understand whether excessive Tfh cells account for the generation of autoantibodies and autoimmunity in *Bach2*^Δ*CD*4^ mice. To this end, we crossed mice carrying *Lox*P-flanked *Bcl6* alleles with *Bach2*^Δ*CD*4^ mice to generate *Bcl6*^fl/+^*Bach2*^Δ*CD*4^ mice. The *Bcl6* heterozygosity was capable to limit the spontaneous and induced GC responses (20). As expected, the *Bcl6* heterozygosity significantly reduced the frequencies of Tfh cells and chronic GC B cells in the MLNs of *Bach2*^Δ*CD*4^ mice at 5 months of age (Figures 7A,B). The expansion of spontaneous GC B cells in the spleens of these mice was also markedly attenuated, although the reduction of Tfh cells was not significant (Figures 7A,B). We then tested whether reduction in the chronic and spontaneous GC responses in *Bcl6*^fl/+^*Bach2*^Δ*CD*4^ mice was accompanied by reduced pathology. We found that the *Bcl6* heterozygosity significantly increased the survival rate and prevented weight loss in *Bach2*^Δ*CD*4^ mice at 5–6 months of age (Figure 7C). The titers of autoantibodies are often parallel with the disease severity and activity in various autoimmune diseases (23, 24). Morbid *Bach2*^Δ*CD*4^ mice contained significantly higher titers of serum anti-dsDNA and anti-ANA antibodies when compared with the normal counterparts (Figure 7D). Although anti-dsDNA and anti-ANA antibodies were easily detected in the sera of *Bcl6*^fl/+^*Bach2*^Δ*CD*4^ mice, their titers were significantly lower than those in morbid *Bach2*^Δ*CD*4^ mice (Figure 7D).

## Discussion

Our study gains new insights into Bach2-dependent control of humoral autoimmunity. Through our cell-type-specific *Bach2*-deletion experiments, we confirm that Bach2 expression in T cells, but not in B cells, is essential for prevention of humoral autoimmunity. Bach2 restrains accumulation of Tfh cells, especially IL-4-producing subset, under chronic antigenic stimulation and steady state. Furthermore, we provide evidences to show that Bach2 prevents humoral autoimmunity, at least in part by inhibiting the generation of pathogenic Tfh cells. More recently, two groups reported that Bach2 limits Tfh cell differentiation in response to acute stimulation of foreign antigens (39, 40). In combination with our results, Bach2 appears to be an important negative regulator during Tfh cell differentiation under various conditions. In addition to Tfh cells, Bach2 instructs the differentiation and effector functions of other T cell subsets including Treg and Th17 cells. Deregulated Treg and Th17 cells play prominent roles in autoimmunity. We observed partial loss of Treg cells in *Bach2*^Δ*CD*4^ mice. It is likely that these mice also harbor pathogenic Th17 cells. These abnormalities may significantly contribute to autoimmunity in *Bach2*^Δ*CD*4^ mice.

CXCR5 is a lineage-defining marker of Tfh cells, and Tfh cell differentiation is initiated by CXCR5 up-regulation (15, 16). Bach2 was reported to directly suppress *Cxcr5* transcription (39). This conclusion was further confirmed in our study. In addition, we found that Bach2 is capable to directly repress *c-Maf*, a key regulator of Tfh cells. c-Maf is up-regulated during Tfh cell differentiation and facilitates the expression of many Tfh-related genes including *Cxcr5, Icos, Pdcd-1*, and *IL-21* (32–34). Loss of c-*Maf* expression in the T cell compartment leads to defective development of Tfh cells in response to both antigen/adjuvant vaccinations and commensal intestinal bacteria (34). Thus, it is conceivable here that *Bach2* deficiency results in aberrant Tfh cell formation mainly *via* up-regulation of c-Maf and CXCR5. Unexpectedly, although ectopic expression of Bach2 was shown to repress Bcl6 expression in differentiated Tfh cells induced by acute immunization with protein antigen (40), Bach2 deletion had no significant effect on Bcl6 mRNA abundance in Tfh cells induced by chronic antigenic stimulation. The different results may be due to different experimental settings.

Bach2 is highly expressed in GC B cells and is required for antibody class switching and blockade of plasma cell differentiation (8, 9). In contrast, Bach2 is gradually down-regulated during Tfh cell differentiation and maintained at low levels in differentiated Tfh cells (Figure 4A). Currently, little is known about the transcriptional control of Bach2 in T cells. BCL6 may be a crucial transcriptional factor that binds to the Bach2 gene locus and suppresses its transcription (Figures 4B,C). The generation and function of Tfh cells are regulated at multiple stages. Bach2 is likely to act mainly at the early phase to limit aberrant Tfh formation. This is evidenced by the observation that *Bach2* directly suppresses c-Maf and Cxcr5 levels in non-Tfh cells, but not in Tfh cells. Low Bach2 levels may be required for Tfh cells to maintain their identity, supported by the recent finding that ectopic overexpression of Bach2 in murine Tfh cells led to a rapid loss of their phenotype and subsequent breakdown of the GC response (40). Thus, the dynamic expression of Bach2 in B cells and T cells could be crucial in instructing proper germinal center responses.

Tfh cells are heterogeneous cell populations, and some Tfh cells are capable of expressing Th1- or Th2-signature cytokines, IFN-γ or IL-4, both of which contribute to the regulation of B cell antibody class switching within GCs. However, the mechanisms regulating IFN-γ/IL-4-producing subset distribution in Tfh cells remain elusive. *Bach2*-deficient Tfh cells are skewed toward the IL-4-secreting subset, subsequently resulting in a greater proportion of IgG1^+^ and IgE^+^ B cells. To our knowledge, Bach2 is the first reported factor that regulates the balance of Tfh subsets. As the GC response evolves, differentiated Tfh cells extinguish IL-21 secretion and switch to IL-4 production (41). The switching may be repressed by Bach2 because Bach2 inhibits IL-4 expression in Tfh cells. Mechanistically, Bach2 directly binds to the regulatory region of *IL-4* to inhibit its transcription. In addition, Bach2 may indirectly suppress *IL-4* transcription *via* down-regulation of Gata3. IL-4-secreting Tfh cells can originate from Th2 cells in response to chronic antigens such as helminth (42). It is possible that Bach2 specifically represses the conversion of Th2 into IL-4-producing Tfh cells in the lymphoid tissues in the presence of continuous antigenic stimulations. Further experiments are needed to test this hypothesis.

Genetic polymorphisms in the *BACH2* gene locus are associated with numerous autoimmune and allergic diseases in human. Our genetics study in mice suggests that humans carrying the genetic variations in the *BACH2* gene may develop autoimmune diseases by inducing excessive pathogenic Tfh cells. Targeting Tfh cells may represent an effective therapeutic strategy for these patients.

## Data Availability

The RNA-seq data reported in this paper have been deposited in the NCBI Gene Expression Omnibus under accession number GEO135087.

## Author Contributions

HZ and QH conducted most of the experiments and analyzed the data. MZ and ZZ conducted RNA-seq analysis. FY and CP performed immunochemistry and ELISA. CH conceived the idea for the project, supervised the study, and wrote the manuscript.

### Conflict of Interest Statement

The authors declare that the research was conducted in the absence of any commercial or financial relationships that could be construed as a potential conflict of interest.

## References

[1] Richer MJ Lang ML Butler NS. T cell fates zipped up: how the Bach2 basic leucine zipper transcriptional repressor directs T cell differentiation and function. J Immunol. (2016) 197:1009–15. 10.4049/jimmunol.1600847 27496973 PMC4978142

[2] Ferreira MA Matheson MC Duffy DL Marks GB Hui J Le Souef P. Identification of IL6R and chromosome 11q13.5 as risk loci for asthma. Lancet. (2011) 378:1006–14. 10.1016/S0140-6736(11)60874-X 21907864 PMC3517659

[3] Jin Y Birlea SA Fain PR Ferrara TM Ben S Riccardi SL. Genome-wide association analyses identify 13 new susceptibility loci for generalized vitiligo. Nat Genet. (2012) 44:676–80. 10.1038/ng.2272 22561518 PMC3366044

[4] International Multiple Sclerosis Genetics Consortium, Wellcome Trust Case Control Consortium Sawcer S Hellenthal G Pirinen M Spencer CC. Genetic risk and a primary role for cell-mediated immune mechanisms in multiple sclerosis. Nature. (2011) 476:214–9. 10.1038/nature10251 21833088 PMC3182531

[5] Cooper JD Smyth DJ Smiles AM Plagnol V Walker NM Allen JE. Meta-analysis of genome-wide association study data identifies additional type 1 diabetes risk loci. Nat Genet. (2008) 40:1399–401. 10.1038/ng.249 18978792 PMC2635556

[6] Morris DL Sheng Y Zhang Y Wang YF Zhu Z Tombleson P. Genome-wide association meta-analysis in Chinese and European individuals identifies ten new loci associated with systemic lupus erythematosus. Nat Genet. (2016) 48:940–6. 10.1038/ng.3603 27399966 PMC4966635

[7] Roychoudhuri R Hirahara K Mousavi K Clever D Klebanoff CA Bonelli M. BACH2 represses effector programs to stabilize T(reg)-mediated immune homeostasis. Nature. (2013) 498:506–10. 10.1038/nature12199 23728300 PMC3710737

[8] Muto A Tashiro S Nakajima O Hoshino H Takahashi S Sakoda E. The transcriptional programme of antibody class switching involves the repressor Bach2. Nature. (2004) 429:566–71. 10.1038/nature02596 15152264

[9] Huang C Geng H Boss I Wang L Melnick A. Cooperative transcriptional repression by BCL6 and BACH2 in germinal center B-cell differentiation. Blood. (2014) 123:1012–20. 10.1182/blood-2013-07-518605 24277074 PMC3924924

[10] Tsukumo S Unno M Muto A Takeuchi A Kometani K Kurosaki T. Bach2 maintains T cells in a naive state by suppressing effector memory-related genes. Proc Nat Acad Sci USA. (2013) 110:10735–40. 10.1073/pnas.1306691110 23754397 PMC3696756

[11] Kim EH Gasper DJ Lee SH Plisch EH Svaren J Suresh M. Bach2 regulates homeostasis of Foxp3+ regulatory T cells and protects against fatal lung disease in mice. J Immunol. (2014) 192:985–95. 10.4049/jimmunol.1302378 24367030 PMC3946995

[12] Yu X Lao Y Teng XL Li S Zhou Y Wang F. SENP3 maintains the stability and function of regulatory T cells *via* BACH2 deSUMOylation. Nat Commun. (2018) 9:3157. 10.1038/s41467-018-05676-6 30089837 PMC6082899

[13] Kuwahara M Ise W Ochi M Suzuki J Kometani K Maruyama S. Bach2–Batf interactions control Th2-type immune response by regulating the IL-4 amplification loop. Nat Commun. (2016) 7:12596. 10.1038/ncomms12596 27581382 PMC5025763

[14] Kuwahara M Suzuki J Tofukuji S Yamada T Kanoh M Matsumoto A. The Menin–Bach2 axis is critical for regulating CD4 T-cell senescence and cytokine homeostasis. Nat Commun. (2014) 5:3555. 10.1038/ncomms4555 24694524 PMC3988815

[15] Crotty S. Follicular helper CD4 T cells (TFH). Ann Rev Immun. (2011) 29:621–63. 10.1146/annurev-immunol-031210-101400 21314428

[16] Vinuesa CG Linterman MA Yu D MacLennan IC Follicular helper T cells. Ann Rev Immun. (2016) 34:335–68. 10.1146/annurev-immunol-041015-055605 26907215

[17] Ueno H Banchereau J Vinuesa CG. Pathophysiology of T follicular helper cells in humans and mice. Nat Immunol. (2015) 16:142–52. 10.1038/ni.3054 25594465 PMC4459756

[18] Vinuesa CG Sanz I Cook MC. Dysregulation of germinal centres in autoimmune disease. Nat Rev Immunol. (2009) 9:845–57. 10.1038/nri2637 19935804

[19] Craft JE. Follicular helper T cells in immunity and systemic autoimmunity. Nat Rev Rheumatol. (2012) 8:337–47. 10.1038/nrrheum.2012.58 22549246 PMC3604997

[20] Linterman MA Rigby RJ Wong RK Yu D Brink R Cannons JL. Follicular helper T cells are required for systemic autoimmunity. J Exp Med. (2009) 206:561–76. 10.1084/jem.20081886 19221396 PMC2699132

[21] Vinuesa CG Cook MC Angelucci C Athanasopoulos V Rui L Hill KM. A RING-type ubiquitin ligase family member required to repress follicular helper T cells and autoimmunity. Nature. (2005) 435:452–8. 10.1038/nature03555 15917799

[22] Vaeth M Eckstein M Shaw PJ Kozhaya L Yang J Berberich-Siebelt F. Store-operated Ca(2+) entry in follicular T cells controls humoral immune responses and autoimmunity. Immunity. (2016) 44:1350–64. 10.1016/j.immuni.2016.04.013 27261277 PMC4917422

[23] Zhang X Lindwall E Gauthier C Lyman J Spencer N Alarakhia A. Circulating CXCR5+CD4+helper T cells in systemic lupus erythematosus patients share phenotypic properties with germinal center follicular helper T cells and promote antibody production. Lupus. (2015) 24:909–17. 10.1177/0961203314567750 25654980

[24] Choi JY Ho JH Pasoto SG Bunin V Kim ST Carrasco S. Circulating follicular helper-like T cells in systemic lupus erythematosus: association with disease activity. Arthritis Rheumatol. (2015) 67:988–99. 10.1002/art.39020 25581113 PMC4450082

[25] Ma CS Deenick EK Batten M Tangye SG. The origins, function, and regulation of T follicular helper cells. J Exp Med. (2012) 209:1241–53. 10.1084/jem.20120994 22753927 PMC3405510

[26] Crotty S. T follicular helper cell differentiation, function, and roles in disease. Immunity. (2014) 41:529–42. 10.1016/j.immuni.2014.10.004 25367570 PMC4223692

[27] Liu X Nurieva RI Dong C. Transcriptional regulation of follicular T-helper (Tfh) cells. Immunol Rev. (2013) 252:139–45. 10.1111/imr.12040 23405901 PMC3579502

[28] Jogdand GM Mohanty S Devadas S. Regulators of Tfh cell differentiation. Front Immunol. (2016) 7:520. 10.3389/fimmu.2016.00520 27933060 PMC5120123

[29] Yu D Rao S Tsai LM Lee SK He Y Sutcliffe EL. The transcriptional repressor Bcl-6 directs T follicular helper cell lineage commitment. Immunity. (2009) 31:457–68. 10.1016/j.immuni.2009.07.002 19631565

[30] Nurieva RI Chung Y Martinez GJ Yang XO Tanaka S Matskevitch TD. Bcl6 mediates the development of T follicular helper cells. Science. (2009) 325:1001–5. 10.1126/science.1176676 19628815 PMC2857334

[31] Johnston RJ Poholek AC DiToro D Yusuf I Eto D Barnett B. Bcl6 and Blimp-1 are reciprocal and antagonistic regulators of T follicular helper cell differentiation. Science. (2009) 325:1006–10. 10.1126/science.1175870 19608860 PMC2766560

[32] Bauquet AT Jin H Paterson AM Mitsdoerffer M Ho IC Sharpe AH. The costimulatory molecule ICOS regulates the expression of c-Maf and IL-21 in the development of follicular T helper cells and TH-17 cells. Nat Immunol. (2009) 10:167–75. 10.1038/ni.1690 19098919 PMC2742982

[33] Kroenke MA Eto D Locci M Cho M Davidson T Haddad EK. Bcl6 and Maf cooperate to instruct human follicular helper CD4 T cell differentiation. J Immunol. (2012) 188:3734–44. 10.4049/jimmunol.1103246 22427637 PMC3324673

[34] Andris F Denanglaire S Anciaux M Hercor M Hussein H Leo O. The transcription factor c-Maf promotes the differentiation of follicular helper T cells. Front Immunol. (2017) 8:480. 10.3389/fimmu.2017.00480 28496444 PMC5406410

[35] Kim CJ Lee CG Jung JY Ghosh A Hasan SN Hwang SM. The transcription factor Ets1 suppresses T follicular helper type 2 cell differentiation to halt the onset of systemic lupus erythematosus. Immunity. (2018) 49:1034–48.e8. 10.1016/j.immuni.2018.10.012 30566881

[36] Peng C Hu Q Yang F Zhang H Li F Huang C. BCL6-mediated silencing of PD-1 ligands in germinal center B cells maintains follicular T cell population. J Immunol. (2019) 202:704–13. 10.4049/jimmunol.1800876 30567732

[37] Hatzi K Nance JP Kroenke MA Bothwell M Haddad EK Melnick A. BCL6 orchestrates Tfh cell differentiation *via* multiple distinct mechanisms. J Exp Med. (2015) 212:539–53. 10.1084/jem.20141380 25824819 PMC4387288

[38] Yusuf I Kageyama R Monticelli L Johnston RJ Ditoro D Hansen K. Germinal center T follicular helper cell IL-4 production is dependent on signaling lymphocytic activation molecule receptor (CD150). J Immunol. (2010) 185:190–202. 10.4049/jimmunol.0903505 20525889 PMC2913439

[39] Geng J Wei H Shi B Wang YH Greer BD Pittman M. Bach2 negatively regulates T follicular helper cell differentiation and is critical for CD4(+) T cell memory. J Immunol. (2019) 202:2991–8. 10.4049/jimmunol.1801626 30971440 PMC6504585

[40] Lahmann A Kuhrau J Fuhrmann F Heinrich F Bauer L Durek P. Bach2 controls T follicular helper cells by direct repression of Bcl-6. J Immunol. (2019) 202:2229–39. 10.4049/jimmunol.1801400 30833348

[41] Weinstein JS Herman EI Lainez B Licona-Limon P Esplugues E Flavell R. TFH cells progressively differentiate to regulate the germinal center response. Nat Immunol. (2016) 17:1197–205. 10.1038/ni.3554 27573866 PMC5030190

[42] Glatman Zaretsky A Taylor JJ King IL Marshall FA Mohrs M Pearce EJ. T follicular helper cells differentiate from Th2 cells in response to helminth antigens. J Exp Med. (2009) 206:991–9. 10.1084/jem.20090303 19380637 PMC2715032

