# Supplementary material for: Bach2 Deficiency Leads to Spontaneous Expansion of IL-4-Producing T Follicular Helper Cells and Autoimmunity
Source: Front Immunol. 2019 Sep 4;10:2050. doi: 10.3389/fimmu.2019.02050 (PMC6737000; doi:10.3389/fimmu.2019.02050)
Supplement: Supplementary file 1 [file Data_Sheet_1.pdf]

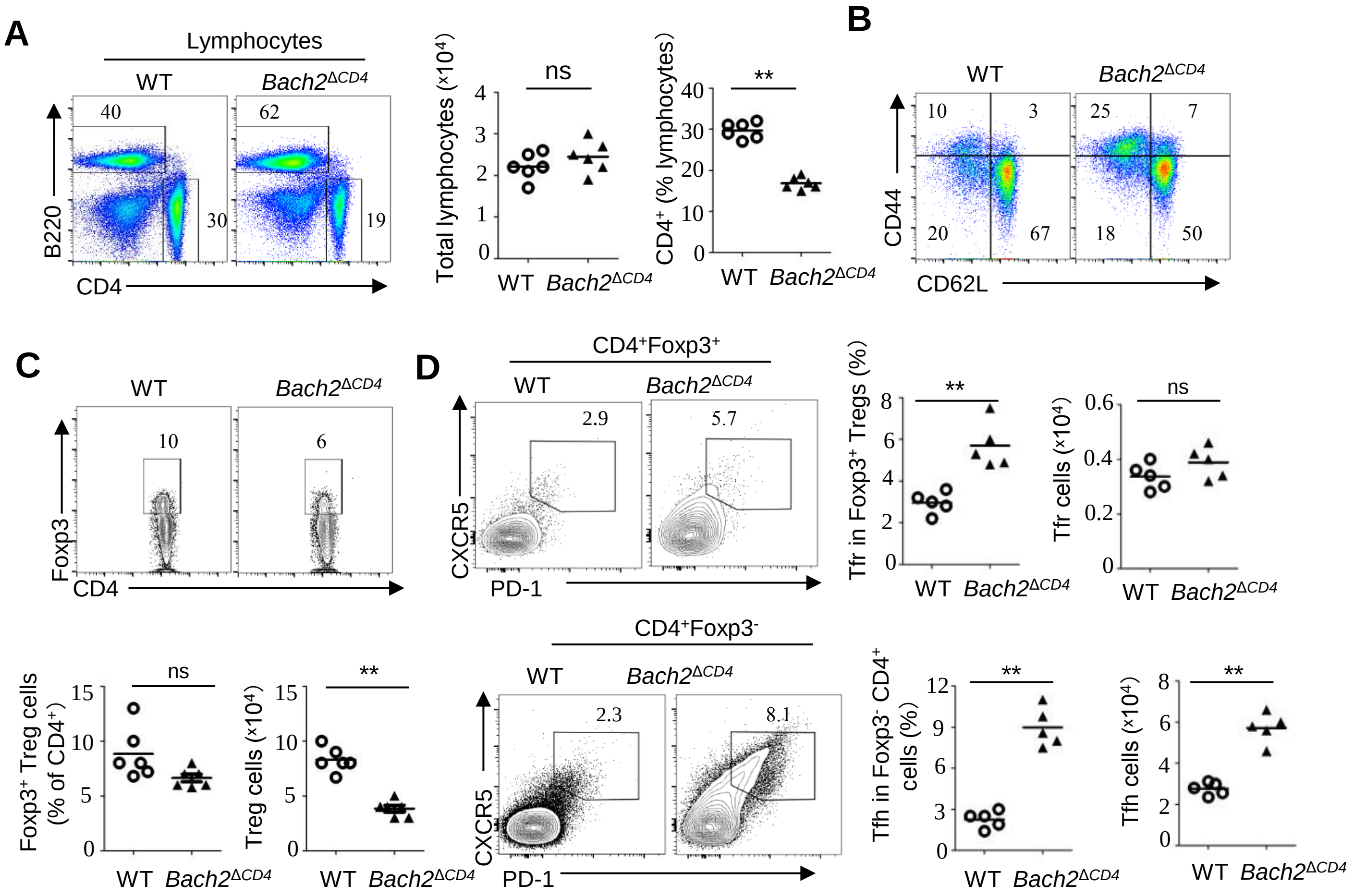

Fig. S1. Expansion of Tfh cells in the MLNs of *Bach2*$^{\Delta CD4}$ mice. Lymphocytes collected from the MLNs of *Bach2*$^{fl/fl}$*Cd4*-cre and WT mice (8-10 weeks old) were subjected for analysis. **(A)** Representative flow cytometry plot of CD4^{+} T cells and B cells gated on the lymphocytes (top). Right: total numbers of lymphocytes, frequencies of CD4^{+} T cells and B cells among lymphocytes. **(B)** Representative flow cytometry plot of CD44 and CD62L expression on CD4 T cells. **(C)** Representative flow cytometry plot, frequency and cell number of Treg cells (gated on CD4^{+} T cells). **(D)** Representative flow cytometry plot, frequency and cell number of CXCR5^{+}PD-1^{+} Tfr (gated on CD4^{+} Foxp3^{+} cells) and CXCR5^{+}PD-1^{+} Tfh (gated on CD4^{+} Foxp3^{-} cells). All data were from at least two independent experiments. Each symbol represents one mouse, and small horizontal lines. ns, not significant and $^{**}P$ <0.01 (two-tailed t-test).

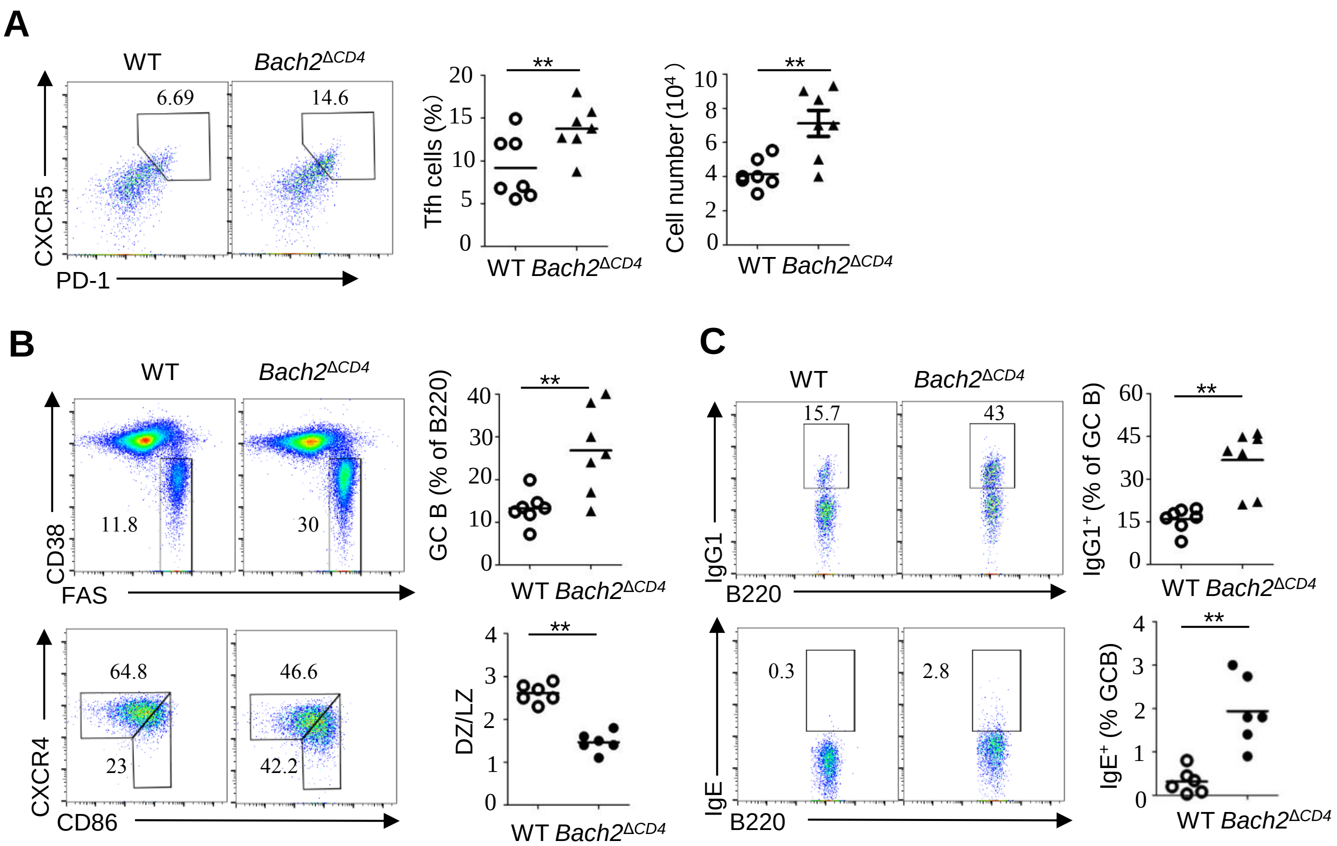

Fig.S2. *Bach2$^{\Delta CD4}$* mice display excessive Tfh cells and aberrant GC B-cells in the Peyer's patches. Lymphocytes collected from the Peyer's patches of *Bach2$^{\Delta CD4}$* and WT mice (8-10 weeks old) were subjected for analysis. **(A)** Representative flow cytometry plot, frequency and cell number of CXCR5^{+}PD-1^{+} Tfh (gated on CD4^{+}Foxp3^{-} cells). **(B)** Representative flow cytometry plot, quantification of frequency of CD38$^{lo/-}$Fas^{+} GC B cells among live B220^{+} cells. **(C)** Representative flow cytometry plot of IgG1^{+} and IgE^{+} cells among GC B cells (left) and their frequencies (right). All data were from at least two independent experiments. Each symbol represents one mouse, and small horizontal lines. All data were from at least two independent experiments. Each symbol represents one mouse, and small horizontal lines. ns, not significant and $^{**}P$ <0.01 (two-tailed t-test).

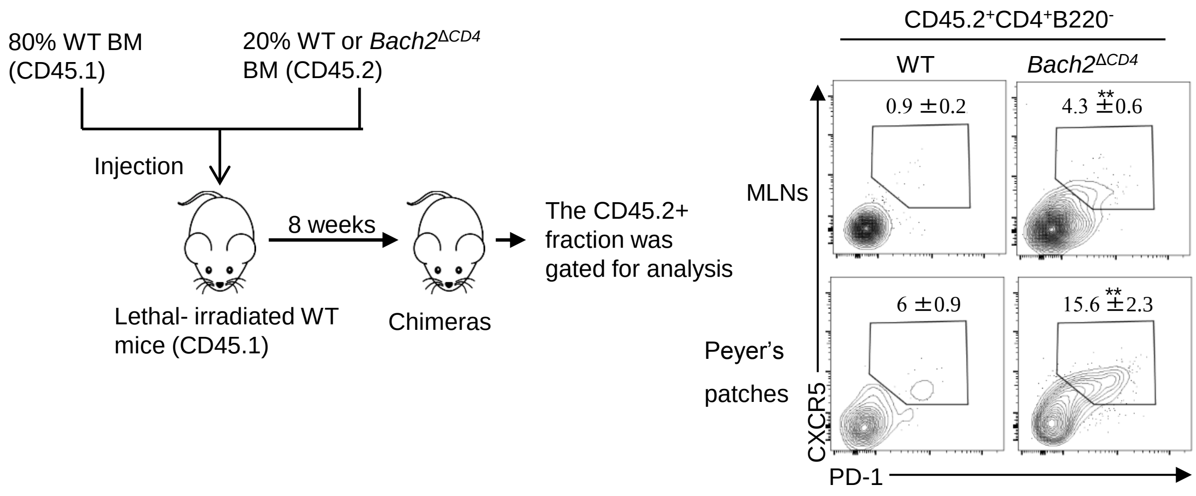

Fig.S3. ***Bach2* deficiency leads to excessive Tfh cells in T-cell intrinsic manner.** The strategy for generation of chimera using mixed bone marrow transplantation (left panel). Representative flow cytometry plot and quantification of frequency of CXCR5$^+$PD-1$^+$ T follicular cells (gated on CD45.2$^+$CD4$^+$B220$^-$ cells). All data were represented as mean ±s.e.m. from three mice. ***P* <0.01 (two-tailed t-test).

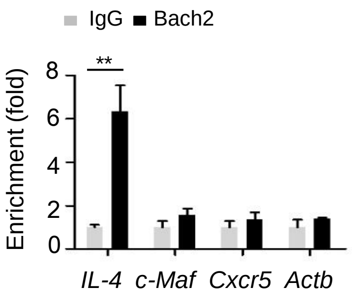

Fig.S4. Bach2 binds to the IL-4 gene in Tfh-like cells. ChIP-qPCR analysis of Bach2 binding at indicated genomic loci in Tfh-like cells. The fold enrichment was calculated relative to IgG. Data are mean ± s.e.m. of two independent experiments. **P <0.01 (two-tailed t-test).

Table S1.

**Primers for RT-qPCR**	
Actb Forward: 5’-TCCGGCACTACCGAGTTATC-3’	Actb Reverse: 5’-GATCCGGTGTAGCAGATCGC-3’
Bcl6 Forward: 5’-AGTCCCCACAGCATACAGAGAT-3’	Bcl6 Reverse: 5’-CCCATTCTCACAGCTAGAATCC-3’
Pdcd1 Forward: 5’-ACCCTGGTCATTCACTTGGG-3’	Pdcd1 Reverse: 5’-CATTTGCTCCCTCTGACACTG-3’
Cxcr5 Forward: 5’-ATGAACTACCCACTAACCCTGG-3’	Cxcr5 Reverse: 5’-TGTAGGGGAATCTCCGTGCT-3’
Stat3 Forward: 5’-CAATACCATTGACCTGCCGAT-3’	Stat3 Reverse: 5’-GAGCGACTCAAACTGCCCT-3’
Stat5a Forward: 5’-CAGATGCAAGTGTTGTATGGGC-3’	Stat5a Reverse: 5’-GCTGGCTCTCGATCCACTG-3’
Maf Forward: 5’-ACTTCGACGACCGCTTCTCG-3’	Maf Reverse: 5’-TCCGCCTCTTCTGCTTCAG-3’
Batf Forward: 5’-ACTTCGACGACCGCTTCTCG-3’	Batf Reverse: 5’-TCCGCCTCTTCTGCTTCAG-3’
Ascl2 Forward: 5’-CGCTGCCCAGACTCATGCCC-3’	Ascl2Reverse: 5’-GCTTTACGCGGTTGCGCTCG-3’
Irf4 Forward: 5’-TCCGACAGTGGTTGATCGAC-3’	Irf4 Reverse: 5’-CCTCACGATTGTAGTCCTGCTT-3’
Lef1 Forward: 5’-AACGAGTCCGAAATCATCCCA-3’	Lef1 Reverse: 5’-GCCAGAGTAACTGGAGTAGGA-3’
Tcf7 Forward: 5’-AGCTTTCTCCACTCTACGAACA-3’	Tcf7 Reverse: 5’-AATCCAGAGAGATCGGGGGTC-3’
Foxp1 Forward: 5’-GGTCTGAG CAAAAAGTAACGGA-3’	Foxp1 Reverse: 5’-CGCACTCTAGTAAGTGGTTGC-3’
Icos Forward: 5’-ATGAAGCCGTACTTCTGCCG-3’	Icos Reverse: 5’-CGCATTTTTAACTGCTGGACAG-3’
Il-4 Forward: 5’-AGATCACGGCATTTTGAACG-3’	Il-4 Reverse: 5’-TTTGGCACATCCATCTCCG-3’
Il-21 Forward: 5’-GGAAGTGCAAACCTCACTATCT-3’	Il-21 Reverse: 5’- TTCATCACAGGACACCCATAAC -3’
**Primers for ChIP-qPCR**	
Maf Forward: 5’-CTCTTCTGCCTGGCTCTTATG -3’	Maf Reverse: 5’-CACCTCGGTCTTGCACTTT’
IL-4 Forward: 5’-ACAGGAACTGAAATGCACAAAG-3’	IL-4 Reverse: 5’-GACAGATGTGACAGGCTGATAG -3’
Cxcr5 Forward: 5’-CTGATGACACAGCACTGG-3’	Cxcr5 Reverse: 5’-CTGACCACGTCTGTCACT -3’
Actb Forward: 5’-TCTTCTTGCAACACCTCCAG-3’	Actb Reverse: 5’-GCCATCCTATCCCAAGCATA -3’
Bach2 pro Forward: 5’-TCCCTCTGCTGTTCCAAAAC-3’	Bach2 pro Reverse: 5’-GCCCGCTTTTATGGCATT -3’
GAPDH Forward: 5’-ACGTAGCTCAGGCCTCAAGA-3’	GAPDH Reverse: 5’-GCTGCGGGCTCAATTTATAG -3’

Table S1. Sequences of primers used in this study.
